# VICO: Ontology-based representation and integrative analysis of Vaccination Informed Consent forms

**DOI:** 10.1186/s13326-016-0062-4

**Published:** 2016-04-19

**Authors:** Yu Lin, Jie Zheng, Yongqun He

**Affiliations:** Unit for Laboratory Animal Medicine, University of Michigan Medical School, Ann Arbor, MI 48109 USA; Department of Microbiology and Immunology, University of Michigan Medical School, Ann Arbor, MI 48109 USA; Center for Computational Medicine and Bioinformatics, University of Michigan Medical School, Ann Arbor, MI 48109 USA; Comprehensive Cancer Center, University of Michigan Medical School, 1301 MSRB III, 1150 W. Medical Dr., Ann Arbor, MI 48109 USA; Department of Genetics, University of Pennsylvania Perelman School of Medicine, Philadelphia, PA 19104 USA

## Abstract

**Background:**

Although signing a vaccination (or immunization) informed consent form is not a federal requirement in the US and Canada, such a practice is required by many states and pharmacies. The content and structures of these informed consent forms vary, which makes it hard to compare and analyze without standardization. To facilitate vaccination informed consent data standardization and integration, it is important to examine various vaccination informed consent forms, patient answers, and consent results. In this study, we report a Vaccination Informed Consent Ontology (VICO) that extends the Informed Consent Ontology and integrates related OBO foundry ontologies, such as the Vaccine Ontology, with a focus on vaccination screening questionnaire in the vaccination informed consent domain.

**Results:**

Current VICO contains 993 terms, including 248 VICO specific terms and 709 terms imported from 17 OBO Foundry ontologies. VICO ontologically represents and integrates 12 vaccination informed consent forms from the Walgreens, Costco pharmacies, Rite AID, University of Maryland College Park, and the government of Manitoba, Canada. VICO extends Informed Consent Ontology (ICO) with vaccination screening questionnaires and questions. Our use cases and examples demonstrate five usages of VICO. First, VICO provides standard, robust and consistent representation and organization of the knowledge in different vaccination informed consent forms, questionnaires, and questions. Second, VICO integrates prior knowledge, e.g., the knowledge of vaccine contraindications imported from the Vaccine Ontology (VO). Third, VICO helps manage the complexity of the domain knowledge using logically defined ontological hierarchies and axioms. VICO glues multiple schemas that represent complex vaccination informed consent contents defined in different organizations. Fourth, VICO supports efficient query and comparison, e.g., through the Description Language (DL)-Query and SPARQL. Fifth, VICO helps discover new knowledge. For instance, by integrating the prior knowledge imported from the VO with a user’s answer to informed consent questions (e.g., allergic reaction question) for a specific vaccination, we can infer whether or not the patient can be vaccinated with the vaccine.

**Conclusions:**

The Vaccination Informed Consent Ontology (VICO) represents entities related to vaccination informed consents with a special focus on vaccination informed consent forms, and questionnaires and questions in the forms. Our use cases and examples demonstrated how VICO could support a platform for vaccination informed consent data standardization, data integration, and data queries.

## Background

Different countries and organizations have various regulations in terms of the requirement of informed consent to vaccination before vaccinating a patient. Signing a vaccination (or immunization) informed consent form is not a federal requirement in the US and Canada. As an exception, the 1976 swine flu immunization program established by the US Federal legislation, included requirements that recipients of the swine flu vaccine be fully informed of the risks and benefits of immunization and that written consent forms be used (42 U.S.C.A.§247b(j)(1)(F) (Supp.1977) [1]. The USA National Childhood Vaccine Injury Act of 1986 (NCVIA - 42 U.S.C. § 300aa-26) requires that the Vaccine Information Statements (VISs) (http://http://www.immunize.org/vis/) must be provided by all public and private vaccination providers to the patient (or parent or guardian) prior to every dose of specific vaccines. Although there is no US federal requirement, the documentation of consent is recommended or required by some states, local health authorities, school authorities, or pharmacies. The immunization protocols from many pharmacies require the signatures of recipients or legal representatives on specific vaccination informed consent forms before vaccination in accordance with local state’s legislation (https://www.pharmacist.com/guidelines-pharmacy-based-immunization-advocacy). The Manitoba Province in Canada provides vaccination informed consent guidelines for routine immunization in accordance with The Public Health Act (C.C.S.M. c. P210) in Canada [2]. Additionally, new laws in Texas were passed in 2013 to allow pregnant minors and minor parents to consent to their own vaccination [3]. Under this circumstance, one can conclude that there must be a variety of vaccination informed consent forms if provided from different sources, such as pharmacies, schools and hospitals. An important question is whether the informed consent for vaccination provides enough adequate information for the patients to understand their risks and benefits prior to their vaccinations. To address this question, an overview and comparison of current informed consent forms are critical.

Due to different policies and vaccination informed consent forms offered by authorities and pharmacies, it is difficult to compare, evaluate, and manage vaccination informed consent procedures and data from different resources. Ontologies, sets of terms and relations that represent entities in a domain and how they relate to each other, support computer-assisted data integration, knowledge management, and new knowledge discovery. Developing a vaccination informed consent ontology that can advance integrating data from different resources may facilitate the research in informed consent and vaccine policy. Therefore, more efficient patient safety management can be achieved.

Based on the principle of ontology reuse, two established ontologies can be re-utilized to ontologically represent vaccination informed consents. One is the community-based Informed Consent Ontology (ICO) [4]. ICO represents the documentations and processes involved in informed consent. ICO aims to support informed consent data integration and reasoning in the clinical research space. Vaccine Ontology (VO) [5, 6] represents licensed vaccines, vaccines in clinical trials, and experimentally verified vaccines in research laboratories. In addition, vaccine-related information including vaccine components, vaccine licenses, vaccine manufacture, vaccination, and vaccination doses are also represented in VO. Both Informed Consent Ontology and Vaccine Ontology are aligned with the upper level Basic Formal Ontology (BFO) [7] and compliant with the Open Biological and Biomedical Ontologies (OBO) Foundry ontology development principles [8]. Built on the same upper ontology, Informed Consent Ontology and Vaccine Ontology can be seamlessly integrated for representing vaccination informed consent.

In this paper, we report the development of the Vaccination Informed Consent Ontology (VICO) by extending Informed Consent Ontology and integrating other OBO Foundry ontologies, such as Vaccine Ontology. The goal of VICO is to represent the vaccination informed consent document, organize vaccination informed consent-related entities, and to establish relations between those entities so that the knowledge of vaccination informed consent can be captured explicitly. The current development focus is to consistently represent different vaccination/immunization informed consent forms, especially the immunization screening questionnaires inside these forms. We then apply VICO to conduct systematic vaccination/immunization informed consent form comparison and patient centered informed consent data query and analysis. We hypothesized that VICO would significantly enhance standard data representation, integration and query of informed consent related to various vaccine immunization procedures. To test the hypothesis, we lay out five major usages of VICO related to the hypothesis, and provide use cases to justify these usages.

## Methods

### Collection of vaccination informed consent forms

Twelve vaccination informed consent forms were collected from the following resources: Costco Pharmacy website [9] (accessed on 01/26/2016), Walgreens Pharmacy website [10] (accessed on 0126/2016), Rite AID website [11] (accessed on 01/26/2016), Manitoba government Public Health division [12] (accessed on 01/26/2016), and the University of Maryland College Park [13] (accessed on 01/26/2016).

### VICO ontology development

VICO was formatted in the Web Ontology Language (OWL2) [14]. The backbone of VICO composed of a portion of Informed Consent Ontology (ICO), a portion of Vaccine Ontology (VO), and VICO specific terms. The workflow of developing VICO includes following four processes:

1. *Extract subset of ICO*. After manually identifying the ICO terms related with vaccination informed consent, the OntoFox tool [15] was used to extract portion of ICO.
2. *Extract subset of VO*. This process includes two steps of extracting portion of VO using two different tools, Ontobee SPARQL endpoint and OntoFox tool. First, all licensed vaccines in USA and Canada were retrieved using the Ontobee SPARQL endpoint (http://www.ontobee.org/sparql) [16] based on the logical axiom defined in VO: *bearer_of some ‘USA licensed vaccine role’*. Next, OntoFox tool was applied to extract a portion of VO that contains all the related terms, logical axioms (classes and relations), definitions, and annotations of each licensed vaccine retrieved from the first step.
3. *Importing ICO and VO subsets to VICO*. The ICO and VO subsets form the backbone of VICO. The importing process is done by using an ‘owl:import’ statement in the VICO owl file. This backbone of VICO with imported ICO and VO subsets can be displayed using the Protégé OWL Editor tool.
4. *Enrich VICO with VICO-specific terms.* We first identify a vocabulary from abovementioned questions, build hierarchy from this vocabulary, and then relate these terms using existing defined relations or VICO specific relations. We establish the logical axioms, so that the questions can be normalized with clear semantics. For example, a question of “have you had received any vaccinations in the past 4 weeks” is related with the “vaccination” term from VO, and is asserted as a subclass of “questions on past vaccination information” VICO term, which has a mother term of ‘question textual entity’ from IAO. Categorizing questions from different forms are mainly based on ontologist’s manual assessment in accordance with hierarchies of question related entities established in existing OBO Foundry ontologies. This way, different forms and questions appeared as different text strings can be related to common existing ontology entities, thus enable automated analyzation of vaccine informed consent form programmatically.

The Protégé OWL Editor version 5.0 beta [17] was used to develop VICO. The HermiT reasoner (http://hermit-reasoner.com/) tool was employed to perform the reasoning over VICO to detect inconsistencies or conflicts.

The VICO Github project (https://github.com/VICO-ontology/VICO) was created to facilitate the project version control and tracking issues.

### VICO evaluation by use cases

Use cases and examples were laid out to evaluate and demonstrate the applications of VICO in supporting different usages. For application evaluation, SPARQL and/or DL languages were often applied. The SPARQL queries were performed using the Protégé SPARQL program or Ontobee’s SPARQL query endpoint (http://www.ontobee.org/sparql) [16]. The DL queries were performed using DL Query plugin of Protégé 5.0 (beta 15) to answer questions from use case 2. Query scripts generated for this project were stored in the Github under the folder:

https://github.com/VICO-ontology/VICO/tree/master/src/SPARQL%20query.

### Ontology source access and license

The VICO is an open source project. The source code including development version and released version are freely available at the URL: https://github.com/vico-ontology/VICO. VICO is released under a Creative Commons 3.0 License.

## Results

### VICO ontology design and top level structure

**Fig. 1:**
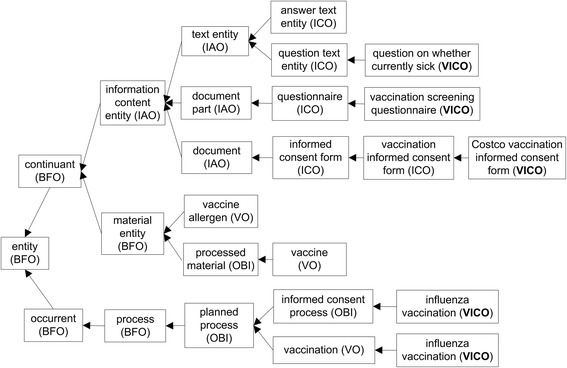
Top level terms and hierarchical structure of VICO. VICO imports many top level terms from VO and ICO and includes VICO-specific terms as exampled with two VICO terms (bold)

VICO is a community-driven ontology that crosses both informed consent domain and vaccine domain. In VICO, we extended Informed Consent Ontology (ICO) and Vaccine Ontology (VO) by adding VICO specific terms representing vaccination informed consent forms (Fig. 1). For example, specific VICO terms were generated to represent the vaccination/immunization informed consent forms from different vaccination providers or governments, such as ‘Costco vaccination informed consent form’ (Fig. 1). Another example is ‘vaccination screening questionnaire’, defined as ‘A questionnaire that contains different questions of a vaccination patient’s health history, allergy history, and current condition, in order to assess the contraindication and precaution for administering a vaccine’ in VICO. This term was generated for representing questionnaire embedded in a vaccination informed consent form. The questions for vaccination patients can be answered by the patients themselves, or their legal representatives, prior to a vaccination procedure. VICO defines these questions in a structured logical manner (Fig. 1).

**Fig. 2:**
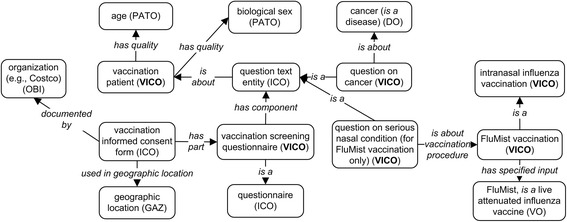
Basic VICO design pattern

The basic VICO ontology design pattern is composed of various entities linked by defined relations as illustrated in Fig. 2. Specifically, before a vaccination process, a vaccination informed consent form is documented by an organization/company (e.g., the company Costco). A vaccination screening questionnaire containing a list of questions is provided for a vaccination patient or his/her legal representative to answer. The ‘documented_by’ is a relation defined by VICO as ‘an object property that represents a relation between a document and an entity that writes, maintains and releases the document’. A specific vaccination consent form is often restricted to be used in a specific geographic location (e.g., Manitoba in Canada or South Carolina in USA). The contents of the questions in the form often cover different topics such as the vaccination patient’s current health status, current treatment, allergic reaction history, past vaccination history, and so on. The vaccination patient’s age and biological sex (female or male) are modelled using PATO term ‘has_quality’ linking the age and biological sex to patient. The vaccination process occurs via a specified vaccination route (e.g., intranasal influenza vaccination for FluMist) (Fig. 2). A question, for example, ‘*question on serious nasal condition’* (asked for FluMist vaccination only) ‘*is about vaccination procedure’* some ‘*FluMist vaccination’*, which is a live attenuated influenza vaccine. A vaccination related question may also be related to a disease (e.g., cancer) or an adverse event process, which has been expressed in VICO using the relation ‘*is about*’ (Fig. 2). Details about ontological modeling of vaccine/vaccination can be found in the previous VO paper [5, 6]. Informed consent representation is introduced in previous ICO paper [4]. By integrating VO/ICO representations and including VICO-specific contents, VICO provides a framework to link vaccination patient, vaccinee quality, vaccine, vaccine quality, vaccination, vaccination targets, informed consent process, informed consent forms, questions, questionnaires, and related information.

As of January 30, 2016, VICO (version 1.0.51) contains 993 terms, including 707 classes, and 92 object properties. VICO includes 243 VICO-specific classes and properties with the “VICO_” prefix, which are new ontology terms not covered in ICO, VO, or any other OBO Foundry ontologies. As shown in the Ontobee VICO statistics page (http://www.ontobee.org/ontostat/VICO), in addition to ICO and VO, VICO also reused terms from other OBO Foundry ontologies such as Information Artifact Ontology (IAO) [18] and Ontology for Biomedical Investigations (OBI) [19], which mainly indirectly imported due to imports of ICO and VO. VICO is deposited in the Ontobee RDF triple store [16], and can be visualized and queried on the Ontobee website: http://www.ontobee.org/ontology/VICO.

### VICO Usages

In general, ontologies can be used in different aspects of knowledge management, including: (i) provide robust and consistent knowledge representation and organization, (ii) integrate prior knowledge, (ii) manage the complexity of domain knowledge, (iv) support efficient query and comparison, and (v) help discover new knowledge. Below we will elaborate how VICO is applied in these above categories of usages.

### Usage 1: Provide robust and consistent knowledge representation and organization

VICO provides a robust and consistent representation of the knowledge of vaccination informed consent in the three major branches: vaccination informed consent form, vaccination screening questionnaire, and questions. The basic relations between these three branches are that a vaccination informed consent form 'has part' a vaccination screening questionnaire that 'has component' many questions. Each of the branches is organized with a hierarchical structure through the ‘is a’ relation. For example, *‘Costco vaccination informed consent form’ ‘is a’ ‘vaccination informed consent form’*. The following example axioms illustrate the relations of terms in the three branches:

1. *‘Costco vaccination informed consent form’: 'has part' some 'questionnaire for Costco vaccination consent'*
2. *‘questionnaire for Costco vaccination consent’: 'has component' some 'question whether allergic to egg'*

All the questions in VICO are organized in a hierarchical structure under ICO term: ‘question textual entity (ICO_0000141)’. VICO modelled 158 questions collected from 12 vaccination informed consent forms. After standardization with newly generated mother terms and hierarchical terms, there are 168 VICO question terms arranged under the IAO term ‘question textual entity’.

**Fig. 3:**
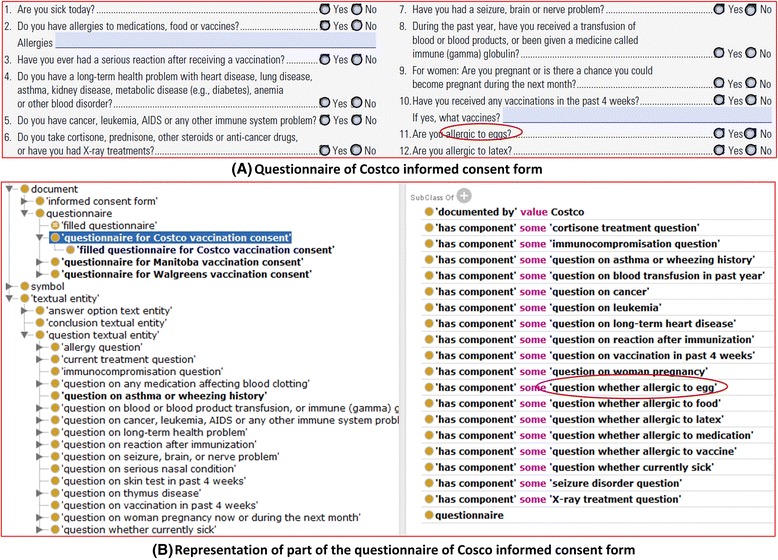
Questionnaire in Costco vaccination informed consent form and its representation in VICO. **a** All the questions shown in the in Costco informed consent form. **b** VICO representation of the questions in the Costco form questionnaire

For robust representation, VICO sometimes breaks down a question with mixed information to more than one specific question. For example, the Costco form lists 12 questions (Fig. 3a). Figure 3b demonstrates how VICO represents these questions, questionnaires and their relations. The question “Do you have allergies to medications, food or vaccines?” is represented in VICO as three VICO question terms: “question whether allergy to food”, “question whether allergy to medication”, and “question whether allergy to vaccine”.

VICO consistently represents linguistic variants of questions with standardized VICO terms. For example, the ‘are you sick today’ and ‘are you well today’ is annotated as term ‘question whether currently sick’. Textual definitions, definition sources, and comments are also provided to ensure clarity and consistency.

The answer to an informed consent question can be “Yes”, “No”, or “unknown”. VICO represents the answer to a question using ‘answer option text entity’ (ICO_0000171), a subclass of ‘textual entity’ (IAO_0000300). We have two ICO terms to represent the “Yes” or “No” answer: ‘yes answer text entity’ (ICO_0000172) and ‘no answer text entity’ (ICO_0000173). VICO created its own “don’t know answer text entity” (VICO_0000006), since in some vaccination informed consent form, “don’t know” was another choice of answer in addition to “Yes” or “No”.

The standard, robust, and coherent representation and organization of the knowledge in the domain of vaccination informed consent are the foundation of the other usages as described below.

### Usage 2: Integrate prior knowledge

VICO provides a way to seamlessly integrate prior knowledge as represented in other ontologies such as the Vaccine Ontology (VO). As a vaccination informed consent ontology, VICO does not focus on the attributes of any licensed vaccines. Fortunately, such information is available in the VO. VO provides informed consent related information for licensed vaccines, including vaccine ingredients, vaccine manufactures, vaccine administration routes, contraindications, etc. Such information is important for interpreting vaccination patients’ answers of informed consent questions. Instead of generating such information from scratch, VICO imported the related information directly from VO.

**Fig. 4:**
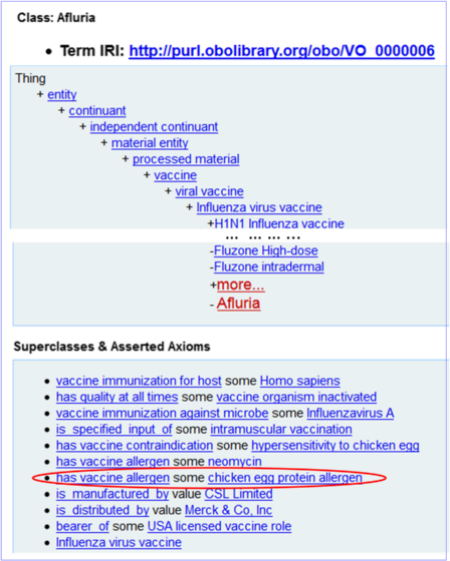
The ontology hierarchy and associated axioms of the Afluria influenza vaccine. The contents are defined in VO and imported to VICO. The figure is a screenshot from Ontobee [16]. The highlighted egg allergen axiom is used for a use case demo detailed later in this paper

As a demonstration of importing prior knowledge from VO, Fig. 4 shows the VO’s representation of a vaccine, Afluria. Afluria is a human influenza viral vaccine licensed for use in the USA. It is an inactivated vaccine against Influenza virus A. It is manufactured by the CSL Limited, and distributed by Merck & Co, Inc. Various characteristics of Afluria were represented using logical axioms in VO. For example, the axiom: *‘has vaccine allergen’ some ‘chicken egg protein allergen’* encodes that the Afluria vaccine contains a trace of chicken egg protein, which is able to induce allergic reaction in certain population. Therefore, the hypersensitivity to chicken egg becomes a contraindication to this vaccine. Another vaccine allergen associated with this vaccine is neomycin. This vaccine is administered via an intramuscular route. The vaccine information has been imported to VICO from VO as detailed in the Methods section. Such importing mechanism provides a valid strategy for VICO to integrate prior knowledge as recorded in VO.

In addition, VICO also imports prior knowledge related to informed consent questions by adding disease information and organizing the questions based on diseases or symptoms. To support the interoperability, the VICO questionnaire questions have been mapped to the Logical Observation Identifiers Names and Codes (LOINC; https://loinc.org/) [20, 21] and semantically linked to the Disease Ontology (DOID) [22], Symptom Ontology (SYMP), Ontology of Adverse Event (OAE), and some newly generated VICO terms for medical procedure or health condition. For example, in VICO, ‘question whether currently sick with a moderate to high fever, vomiting/diarrhea’ is linked to SYMP:‘fever’ and DOID:‘diarrhea’ through the IAO relation ‘is_about’. Another example is “allergy question”, which is related to DOID term “hypersensitivity reaction type I disease” using “is_about” relation. Likewise, “question whether allergy to food” is about “food allergy” (DOID). This way, the VICO bridges questions with existing terms and prior knowledge from these other ontologies.

### Usage 3: Manage the complexity of domain knowledge

The complexity of the vaccination informed consent lies in the complex relations between vaccination patients, patient attributes (e.g., age and gender), vaccine, vaccine attributes, and different levels of question complexity. Such complexity is managed in VICO by generating and following a concise and valid design pattern (Fig. 2) that semantically link all the related classes together. The linkages among different classes are established with solid object properties (i.e., relations).

The complexity of the vaccination informed consent is also reflected by the fact that various vaccination informed consent forms exist for different purposes and are used in different locations. For example, in Rite Aids, different informed consent forms are distributed in different states: South Carolina, North Carolina, California, and other states. Informed consent forms for kids are often different with those for adults, or college students. Although many informed consent forms are generally applied for different vaccinations, in our corpus of vaccination informed consent forms, four different informed consent forms are all about Flu vaccination: University of Maryland College Park injectable influenza vaccination informed consent, University of Maryland College Park Flumist vaccination informed consent, Rite Aid injectable flu vaccination informed consent in South Carolina, and Manitoba Seasonal Influenza and Pneumococcal vaccination informed consent form.

The management of these complex forms and questions in VICO relies on the consistent and robust representation and organization (see Usage 1), and the use of logical axioms to clearly lay out the differences. For example, the relation between a form and its source is clearly defined using the relation ‘documented_by’ as exemplified here: *‘Costco vaccination informed consent form’: ‘documented by’ value Costco*. Another example axiom to link a form with a purpose is here: *‘UM College Park flumist vaccination informed consent’: ‘is about vaccination procedure’ some ‘Flumist vaccination’*. The ‘Flumist vaccination’ is then linked to the influenza vaccine Flumist with another axiom. Compared to different schemas representing various vaccination informed consent contents from different organizations, VICO is unique in that it glues multiple schemas together using well-organized ontological representations.

### Usage 4. Support efficient query and comparison

Based on the consistent and well-organized VICO representation and organization of complex knowledge, we can easily browse in an ontology editor or web ontology browser, or perform SPARQL queries against the VICO to more specifically compare the questions in different forms. SPARQL is an Resource Description Framework (RDF) query language able to retrieve ontology data stored in the RDF format [23]. These ontology browsing and queries can be used to compare questions from different forms.

**Fig. 5:**
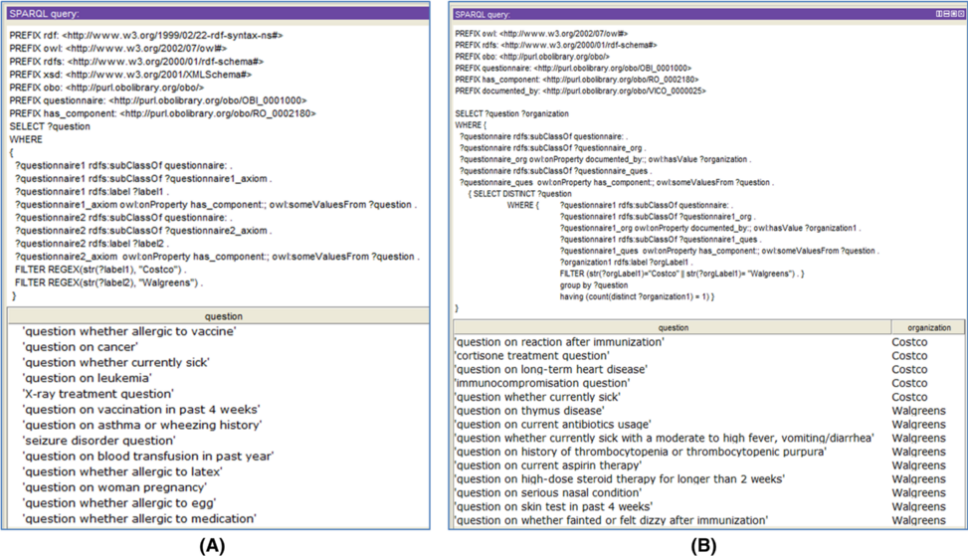
SPARQL query of shared and different questions in Costco and Walgreens vaccination informed consent forms. **a** Query common questions. **b** Query different questions. The two screenshots show the query executions and results generated using the Protégé OWL editor

For example, SPARQL queries were used to compare Costco and Walgreen vaccination informed consent forms. Figure 5 shows how a SPARQL query can be used to identify the common (Fig. 5a) and different (Fig. 5b) questions raised in Costco and Walgreen vaccination informed consent forms. In this SPARQL, we used an object property ‘documented by’ that represents the relation between an informed consent form and an organization (e.g., pharmacy) (Fig. 5b). Our comparative analysis using SPARQL queries found 39 unique questions listed in Walgreens and Costco vaccination informed consent forms (Table 1). Among these questions, 12 questions are shared by both forms, six questions are listed by Costco form only, and 21 questions are unique to the Walgreen form. Compared to the Costco form, four more questions are listed in the Walgreens form. Note that Walgreens unique question “question on whether fainted or felt dizzy after immunization” (question #19) is the subclass of Costco unique question “question on reaction after immunization” (question #18) in VICO. Similarly, “question whether currently sick” (question #13) used by Costco is the parent term of “question whether currently sick with a moderate to high fever, vomiting/diarrhea” (question #21) used by Walgreens. These observations reveal that although both Costco and Walgreens ask the adverse events after vaccination, Walgreens asks more specific and narrower questions.

**Table 1:** Comparison of specific questions listed in Walgreens and Costco vaccination informed consents

Number	Question	Walgreens/Costco
1	question on vaccination in past 4 weeks	Walgreens; Costco
2	question on blood transfusion in past year	Walgreens; Costco
3	question whether allergic to vaccine	Walgreens; Costco
4	question on asthma or wheezing history	Walgreens; Costco
5	question on leukemia	Walgreens; Costco
6	question whether allergic to medication	Walgreens; Costco
7	seizure disorder question	Walgreens; Costco
8	question on cancer	Walgreens; Costco
9	question whether allergic to egg	Walgreens; Costco
10	X-ray treatment question	Walgreens; Costco
11	question whether allergic to latex	Walgreens; Costco
12	question on woman pregnancy	Walgreens; Costco
13	question whether currently sick	Costco
14	question on long-term heart disease	Costco
15	cortisone treatment question	Costco
16	immunocompromisation question	Costco
17	question whether allergic to food	Costco
18	question on reaction after immunization	Costco
19	question on whether fainted or felt dizzy after immunization	Walgreens
20	question on TB skin test in past 4 weeks	Walgreens
21	question whether currently sick with a moderate to high fever, vomiting/diarrhea	Walgreens
22	question on serious nasal condition	Walgreens
23	question on high-dose steroid therapy for longer than 2 weeks	Walgreens
24	question on thymus disease	Walgreens
25	question on current aspirin therapy	Walgreens
26	question on current antibiotics usage	Walgreens
27	question on history of thrombocytopenia or thrombocytopenic purpura	Walgreens
28	question on CSF leak	Walgreens
29	question on asplenia	Walgreens
30	question on azathioprine or 6-mercaptopurine usage	Walgreens
31	question on cochlear implant	Walgreens
32	question on high-dose methotrexate usage	Walgreens
33	question on home infusion	Walgreens
34	question on weekly injection	Walgreens
35	question on weekly injection of adalmumab	Walgreens
36	question on weekly injection of etanercept	Walgreens
37	question on weekly injection of infliximab	Walgreens
38	question whether recieving aspirin-containing therapy	Walgreens
39	question on current anti-malarial medication	Walgreens

The questions asked are typically consistent with patient requirements before vaccinations. For example, the “question on current aspirin therapy” (question #25 in Table 1) is a question specifically for the vaccine FluMist Quadrivalent, asked by Walgreens. To investigate more on why Walgreens asks these vaccine-specific questions, we examined the package insert document for FluMist Quadrivalent. The contraindication section of the FluMist Quadrivalent package insert document says: “Concomitant aspirin therapy in children and adolescents” [24]. This contraindication statement indicates that a patient with concomitant aspirin therapy cannot be vaccinated with the FluMist Quadrivalent vaccine. This information provides the solid reason why the Walgreens form asks whether a patient is taking aspirin.

### Usage 5: Help discover new knowledge

The consistent representation, robust organization, and prior knowledge integration of informed consent forms make VICO a useful platform for new knowledge discovery based on patients’ answers of a questionnaire.

Based on a patient’s answers to informed consent questions, we will demonstrate with a use case on how VICO and OWL-based technologies can be used to discover whether a patient can or cannot be vaccinated with a vaccine containing a trace of egg protein. This use case is related to vaccine contraindication (e.g., allergic reaction to chicken egg), a rare condition in a recipient that increases the risk for a serious adverse reaction. Ignoring contraindications can lead to dangerous vaccine adverse reactions. As shown in Fig. 3, the Afluria influenza vaccine has the contraindication of ‘hypersensitivity to chicken egg’ since it has an egg allergen. Therefore, vaccination of egg-allergic patients with a vaccine (e.g., Afluria) that has an egg allergen is currently not recommended [25].

**Fig. 6:**
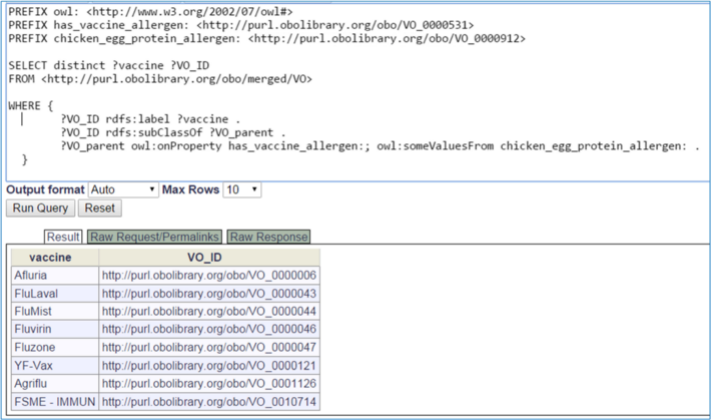
SPARQL query of vaccines that have egg protein allergen. This query was performed using both the Protégé 5.0.0 SPARQL Query plug-in and the Ontobee SPARQL query website (http://www.ontobee.org/sparql/). The SPARQL query codes are available in the Github repository. This figure is a screenshot of the query execution and results using the Protégé SPARQL program

Basically, this use case contains two-steps: *Step 1: Find if a vaccine contains egg allergen, and Step 2: Find if a patient is allergic to egg.* If the answers to both questions are positive, this patient cannot be administered with the vaccine found in step 1. For the first step, we developed a SPARQL query to identify vaccines that have restriction on egg allergic reaction (Fig. 6). As a result, eight licensed vaccines were identified as vaccines containing egg protein allergen. This step is essentially to query the prior knowledge that is imported from VO to VICO.

**Fig. 7:**
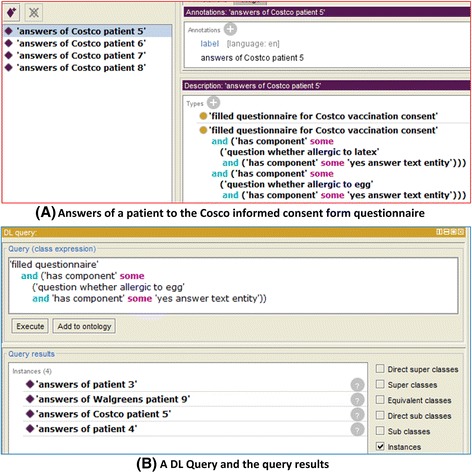
DL query for patients who are allergic to egg. **a** Example of the answers of a Costco patient’s answers to questionnaire. **b** A DL query and its results. The DL query was performed using the Protégé OWL editor

To implement the second step in our sandbox demonstration, first we ontologically represented a patient’s answers to the questions in a filled informed consent form. In this sandbox use case study, we hypothetically created twelve patients’ answers to the questions in the Costco vaccination informed consent form, the Walgreens vaccination informed consent form, or the Manitoba informed consent form respectively. All the answers were instantiated as VICO’s instance data (owl:NamedIndividual). As we mentioned before, in VICO, a patient’s answers to a question is represented as a component of a filled questionnaire instance (Fig. 7). For example, in a Costco vaccination informed consent form, a patient’s ‘No’ answer to the question ‘whether the patient is allergic to egg’ is represented as:

*‘filled questionnaire for Costco vaccination consent’*

*and (‘has component’ some (‘question whether allergic to egg’ and (‘has component’ some ‘no answer text entity’)))*

After the patients’ answers to the informed consent questions were transformed in VICO’s representation, we then performed queries to find out the patient who may be in the danger of trigger a serious egg allergic reaction if given a specific vaccine, e.g., Afluria. In Fig. 7, an OWL Description Logics (DL) query written in the Manchester OWL syntax, a user-friendly syntax for OWL DL [26], was shown and executed using DL Query Tab in the Protégé OWL editor [27]. Out of these twelve patients’ answers, we identified four patients (one Costco patient, one Walgreens patient, and two Manitoba patients) who answered that they were allergic to egg proteins. Therefore, these patients may not be recommended for vaccination with those vaccines containing egg allergen (Fig. 7).

This simplified use case demonstrated how VICO can utilize, integrate prior knowledge from VO (Usage 2), and discover new knowledge (Usage 5) that the patient cannot be vaccinated with a vaccine that contains the chicken egg allergen (e.g., Aflura). In addition, this use case queries patients’ answers (Usage 4) from three different forms in a consistent way, demonstrating the advantages of consistent representation and organization of complex information in the vaccination informed consent forms (see Usages 1 and 2).

## Discussion

This paper introduces the development and application of a Vaccination Informed Consent Ontology (VICO). VICO represents 12 vaccination informed consent forms and corresponding screening questionnaires from different organizations, and over 150 questions in these forms. The top level hierarchical structure and general VICO design pattern are represented, followed by the description of five usages of VICO with examples and use cases.

VICO was developed by following the best practice and recommended strategy of ontology reusing [15]. Instead of coding everything from scratch, we imported related terms from both Vaccine Ontology (VO) and Informed Consent Ontology (ICO) into VICO. The VO includes the information of licensed human vaccines in different countries and related vaccine characteristics. ICO is an ontology representing informed consent that may be applied in broader areas (e.g., clinical trials, clinical research) than vaccine immunization. By importing related terms from these two BFO-based ontologies, VICO demonstrates a seamless integration of existing ontologies. On top of the imported terms, VICO can then focus on the representation of more specific entities such as vaccination informed consent forms from different pharmacies and authorities. As demonstrated in the Results section, VICO has also provided a solution to map vaccination consent questions to the commonly used LOINC standard and generate logical axioms to directly link questions to the diseases shown in the Disease Ontology (DOID). Such a development strategy has been proven successful in our VICO development.

The VICO ontology can not only be used to support Semantics Web applications, but they are also applicable to support relational database systems. One primary reason is that relational database schemas also need consistent, structured, and computer-understandable representation of the informed consent forms, questions, and the relations among questions, vaccines, and patients. VICO standardizes these terms with logical and textual definitions and consistently represents the relations among these terms. VICO also efficiently includes prior knowledge. VICO provides an integrative framework to well represent organize the complexity of different levels of information and knowledge. These usages are also needed for a typical relational database system to occupy. It would be very difficult to hard code all the information, relations, and prior knowledge in software code or relational database. By incorporating VICO to a relational database system, such a system will obtain all the ontology benefits.

VICO is also able to support the integration of different relational databases that most likely use different database schemas. The 12 vaccination informed consent forms collected in our study can be matched to multiple relational database schemas from different organizations. Assuming we want to compare and integratively query the data from these organizations, it is almost impossible to do with relational databases since each organization very likely does not know the database schemas from other organizations. However, with the support of VICO, such a task can be done efficiently as shown in our paper. If each database schema understands the VICO contents, VICO can indeed serve as a hub system that makes different relational database schemas understand each other. This is another reason why VICO can support relational database system.

The Semantic Web formats (e.g., RDF and OWL) provide an inherent capability of reasoning. One example of such reasoning can be found in the reference [28]. Our ontology provides a foundation for reasoning. New rules and equivalent classes can be added to the ontology to support reasoning. The OWL ontology is established based on the RDF technology. Individual RDF triple stores may exist independently and lack an appropriate way to communicate with each other. An ontology provides an effective way to address such a silo issue and semantically link different RDF triple store data to support better data integration and queries.

Linguistic polishing has been used in our process of mapping questions and assigning ontology IDs. However, linguistic polishing cannot achieve many features that we would like to gain by representation in ontology. For example, linguistic polishing does not provide: (i) a hierarchy of questions, (ii) relations of questions to diseases, and (iii) classification of the questions based on diseases and symptoms. Different questions may be associated with the same diseases and concerns. Different questionnaires may have different questions that are related to different vaccines and vendors. These ontological strategy is flexible and extensible to address these questions. In addition, ontology can easily reuse existing knowledge represented in other ontologies (e.g., VO). Most of these use cases cannot be achieved by simple question mapping and intersection of two sets of questions, and their analyses require logical axioms specifically defined in the ontology.

Our VICO usage demonstration makes it feasible to develop electronic interoperable vaccination informed consent forms, e.g., by building up a list of questions for generating the questionnaire in the form. Furthermore, it is now possible for vaccine recipients or their legal representatives to sign electronic informed consent forms. With advantage of sharing Electronic Health Record (EHR), patient’s allergic history, treatment history, and severe adverse event history will be integrated into pharmacy’s IT system, software programs can be developed to automate the screening procedure before recipient or his/her legal representative sign the informed consent form. Clinical decision making system can help to prevent avoidable contraindications prior to vaccination. Compared to an electronic information management system without ontology support, the usage of VICO for supporting an electronic vaccination informed consent management system has many advantages. First, acting as a separate middle layer from the functionalities of the management system, the VICO ontology can be easily updated without additional and costly software engineering work. Secondly, the ontology layer makes it possible to easily perform decision support. Lastly, different informed consent data can be seamlessly integrated together using the same data representation ontology, supporting data integration.

To make VICO useful in practice, there are still several issues to solve. Given the power of our VICO-based SPARQL or a DL queries, in reality, it may be impossible for a clinical professional to type in a SPARQL or a DL query. To bypass the difficulty of using DL or SPARQL, it is possible to develop template-based and natural language-implemented query capability to easily query ontologies [29–31]. A user-friendly query web interface can be provided to support convenient search for those who do not know any programming language. One closely related work is the usage of ontologies for adaptive questionnaires for clinical risk assessment [32, 33]. Adaptive questionnaires are able to dynamically modify the behavior of the structure of the questionnaire in response to user interaction. The context-sensitive adaptation approach can use an ontology as the basic for robust adapted information collection and patient risk assessment [32]. It is possible to use VICO as the ontological basis for adaptive questionnaire formation in an interactive vaccination informed consent management system. To make VICO usable in practice, it is important to link VICO to existing standards and ontologies such as LOINC and DOID, which has been incorporated in our study. Furthermore, we will also need to convince pharmacies and governments who perform or monitor the vaccination procedures with more complete information in the ontology and empirical examples.

VICO-based vaccination informed consent system may be linked to computerized immunization information systems (IIS, or called immunization registries) that are developed to collect and consolidate vaccination data from multi health-care providers, generate automatic notifications, and assess vaccination coverage. Such IIS have been widely established in the US [34]. For example, KSWebIZ Kansas Immunization Registry is a web-based statewide immunization registry that provides a centralized birth to death database of complete and accurate immunization records for all Kansas residents (https://www.contactkswebiz.info/). It would be ideal to eventually link electronic informed consent data to IIS directly, and VICO would facilitate such an effort.

In the future, VICO can also be expanded to cover the information related to vaccine adverse events. Typically, such information is included in the VIS, and the patients need to be notified of the VIS before their consent. Instead of the plain text described in the Vaccine Information Statements (VIS) documents, an Ontology of Vaccine Adverse Event (OVAE) was recently developed to represent various adverse events for each licensed vaccine [35]. OVAE will be imported into VICO, which allows recipients better understanding of possible side effects of vaccinations prior to vaccination, therefore, enhances the informed consent process.

In addition, the original methods identified in this study can be applied to represent informed consent forms in other domains of research (e.g., biobanking [36]). Interoperability strategies applied in this study can be incorporated into existing electronic health records or decision support systems as some stage in the future.

It is noted that current study is still at the prototype stage to prove the rationale and feasibility of applying ontology to solve the issue of data and query disintegration in the area of vaccination inform consents from different vendors and agents. For the current stage of development, manual efforts with software supports have been used to design the ontology, process the data, and implement the use cases. For real usage, automatic computational systems such as natural language processing text mining programs can further be used to improve the efficiency of informed consent content processing. Overall, such a strategy is promising to be used in real clinical setting.

## Conclusions

VICO ontologically represents various entities related to vaccination informed consent including vaccination informed consent forms, various questions in questionnaires, and answers to those questions. Current VICO represents the contents of 12 vaccination informed consent forms from pharmacies, a collage health center, and the government of Manitoba, Canada. Our SPARQL and OWL DL queries demonstrated that VICO could be used as a standard platform for consistently and systematically representing vaccination informed consent form questions, linking question answers to vaccine attributes, identifying potential vaccine contraindications, and enforcing safe vaccination procedures.

## Abbreviations

AE: adverse event
BFO: Basic Formal Ontology
DL query: Description Logics query
DOID: Disease Ontology
IAO: Information Artifact Ontology
ICO: Informed Consent Ontology
LOINC: Logical Observation Identifiers Names and Codes
NCBO: The National Center for Biomedical Ontology
OAE: Ontology of Adverse Events
OBI: Ontology for Biomedical Investigations
OBO: The Open Biological and Biomedical Ontologies
OVAE: Ontology of Vaccine Adverse Events
OWL: Web Ontology Language
RDF: Resource Description Framework
SPARQL: SPARQL Protocol and RDF Query Language
VAE: Vaccine Adverse Event
VICO: Vaccination Informed Consent Ontology
VIOLIN: Vaccine Investigation and Online Information Network
VO: Vaccine Ontology
